# Supplementary material for: Unlocking Bevacizumab’s Potential: rCBVmax as a Predictive Biomarker for Enhanced Survival in Glioblastoma IDH-Wildtype Patients
Source: Cancers (Basel). 2023 Dec 28;16(1):161. doi: 10.3390/cancers16010161 (PMC10778147; doi:10.3390/cancers16010161)
Supplement: Supplementary file 1 [file cancers-16-00161-s001.zip › cancers-2796594-supplementary.pdf]

Supplementary Material

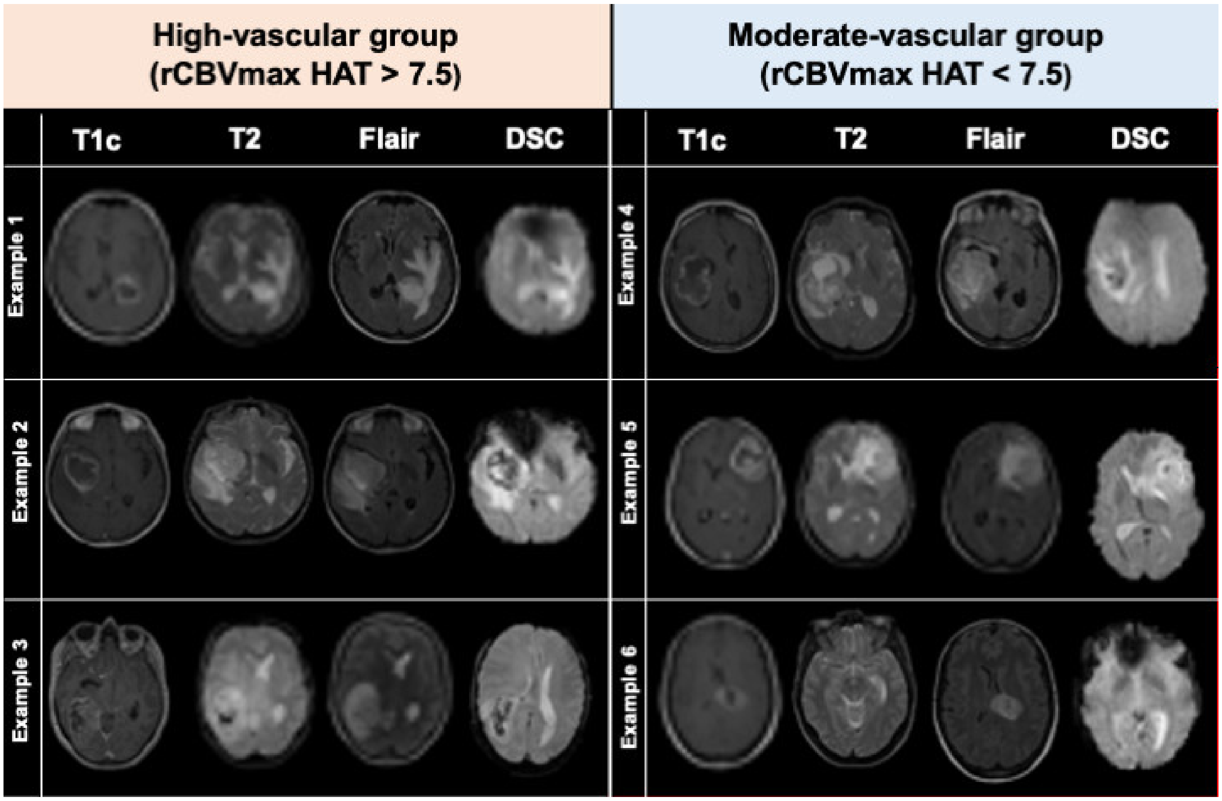

Figure S1. Sets of MRI examples from three patients included in the low-vascular group and three for the high-vascular group.
